# (2*R*,6*S*)-*tert*-Butyl 2-(benzhydryl­carbamo­yl)-6-methyl­morpholine-4-carboxyl­ate

**DOI:** 10.1107/S1600536811017764

**Published:** 2011-05-20

**Authors:** Haiyang Wang, Guangxin Xia, Xuejun Liu, Jingkang Shen

**Affiliations:** a Shanghai Institute of Materia Medica, Chinese Academy of Sciences, Shanghai 201203, People’s Republic of China; b Central Research Institute, Shanghai Pharmaceutical Group Co. Ltd, 555 Zuchongzhi Road, Shanghai 201203, People’s Republic of China

## Abstract

The title compound, C_24_H_30_N_2_O_4_, was obtained by the reaction of (2*R*,6*S*)-4-(*tert*-but­oxy­carbon­yl)-6-methyl­morpho­line-2-carb­oxy­lic acid with diphenyl­methanamine in dimethyl­formamide solution. The morpholine ring is in a chair conformation. In the crystal, weak inter­molecular C—H⋯O hydrogen bonds link mol­ecules into chains along the *b* axis.

## Related literature

For a review of the biological relevance and synthesis of C-substituted morpholine derivatives, see: Wijtmans *et al.* (2004 ▶). For applications of morpholine derivatives as drugs, see: Dando & Perry (2004 ▶); Hajos *et al.* (2004 ▶); Hale *et al.* (1998 ▶); Versiani *et al.* (2002 ▶). For agrochemical fungicides and bactericides containing a morpholine skeleton, see: Dieckmann *et al.* (1993 ▶). For applications of morpholines as chiral auxiliaries in asymmetric synthesis, see: Dave & Sasaki (2004 ▶); Enders *et al.* (1994 ▶).
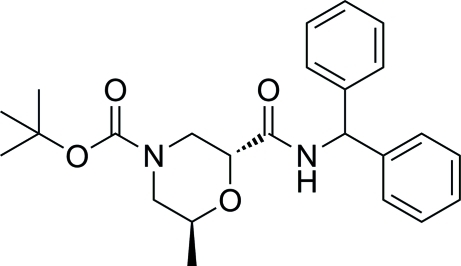


## Experimental

### 

#### Crystal data

C_24_H_30_N_2_O_4_
*M* *_r_* = 410.50
Monoclinic, 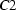

*a* = 27.248 (4) Å
*b* = 5.8241 (8) Å
*c* = 14.275 (2) Å
β = 94.192 (3)°
*V* = 2259.3 (5) Å^3^
*Z* = 4
Mo *K*α radiation
μ = 0.08 mm^−1^
*T* = 293 K
0.37 × 0.24 × 0.16 mm

#### Data collection

Bruker SMART APEX CCD diffractometer
5956 measured reflections
2310 independent reflections
1934 reflections with *I* > 2σ(*I*)
*R*_int_ = 0.110

#### Refinement

*R*[*F* ^2^ > 2σ(*F* ^2^)] = 0.048
*wR*(*F* ^2^) = 0.109
*S* = 0.96
2310 reflections
279 parameters
1 restraint
H atoms treated by a mixture of independent and constrained refinement
Δρ_max_ = 0.15 e Å^−3^
Δρ_min_ = −0.23 e Å^−3^

### 

Data collection: *SMART* (Bruker, 2002 ▶); cell refinement: *SAINT* (Bruker, 2002 ▶); data reduction: *SAINT*; program(s) used to solve structure: *SHELXS97* (Sheldrick, 2008 ▶); program(s) used to refine structure: *SHELXL97* (Sheldrick, 2008 ▶); molecular graphics: *SHELXTL* (Sheldrick, 2008 ▶); software used to prepare material for publication: *SHELXTL*.

## Supplementary Material

Crystal structure: contains datablocks I, global. DOI: 10.1107/S1600536811017764/cv5082sup1.cif

Supplementary material file. DOI: 10.1107/S1600536811017764/cv5082Isup2.cdx

Structure factors: contains datablocks I. DOI: 10.1107/S1600536811017764/cv5082Isup3.hkl

Supplementary material file. DOI: 10.1107/S1600536811017764/cv5082Isup4.cml

Additional supplementary materials:  crystallographic information; 3D view; checkCIF report

**Fig. 1.**
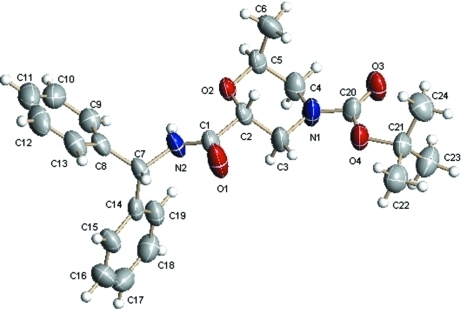
View of (I) showing the atomic numbering. Displacement ellipsoids are drawn at the 50% probability level.

Enhanced figure: interactive version of Fig. 1

## Figures and Tables

**Table 1:** Hydrogen-bond geometry (Å, °)

*D*—H⋯*A*	*D*—H	H⋯*A*	*D*⋯*A*	*D*—H⋯*A*
C19—H19⋯O1^i^	0.93	2.54	3.332 (4)	144

Symmetry code: (i) 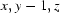.

**Fig. 2.**
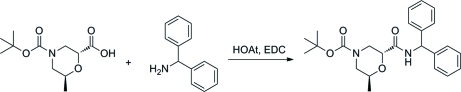
Schematic representation of the synthesis.

## supplementary crystallographic information

### Comment

Morpholines are an important class of heterocyclic compounds found in many naturally occurring or synthetically organic molecules (Wijtmans *et al.*, 2004). Especially, the morpholine moiety has found widespread use in medicinal chemistry, and many drugs contain this subunit. For example, antidepressant drug Reboxetine (Hajos *et al.*, 2004; Versiani *et al.*, 2002), Aprepitant in combination with other agents to prevent and control nausea and vomiting caused by chemotherapy (Dando & Perry, 2004; Hale *et al.*, 1998). The morpholine skeleton is also used to construct a number of agrochemical fungicides and bactericides, such as Fenpropimorph and tridemorph (Dieckmann *et al.*, 1993). Furthermore, morpholines have been applied as chiral auxiliaries in asymmetric synthesis (Dave & Sasaki, 2004; Enders *et al.*, 1994). Herewith we report the crystal structure of the title compound (I).

In (I) (Fig. 1), the morpholine ring is in a chair conformation. Weak intermolecular C—H···O hydrogen bonds (Table 1) link the molecules related by translation along axis *b* into chains.

### Experimental

The schematic representation of the synthesis is given in Fig. 2. To a solution of EDC (125 mg, 0.54 mmol) and HOAt (74 mg, 0.54 mmol) in DMF (2 ml) was added diphenylmethanamine (82 mg, 0.45 mmol) and (2*R*,6S)-4-(*tert*-butoxycarbonyl)-6-methylmorpholine-2-carboxylic acid (132 mg, 0.54 mmol) and the mixture stirred at room temperature overnight. The mixture was then partitioned between EtOAc and water. The organic layer was then washed successively with saturated aqueous sodium bicarbonate, brine and then dried (MgSO4). The solution was evaporated to dryness *in vacuo* and the residue purified by flash column chromatography to give the title compound (124 mg) as a colourless solid. Crystals suitable for X-ray structure analysis were obtained by slow evaporation of a solution in EtOAc at room temperature.

### Refinement

C-bound H atoms were placed in geometrically idealized positions (C—H = 0.93–0.98 Å) and constrained to ride on their parent atoms with *U*_iso_(H) = 1.2–1.5 *U*_eq_(C). Atom H2A was located on difference map and isotropically refined. In the absence of any significant anomalous scatterers in the molecule, attempts to confirm the absolute structure by refinement of the Flack parameter in the presence of 1917 sets of Friedel equivalents led to an inconclusive value of 10 (10). Therefore, the Friedel pairs were merged before the final refinement and the absolute configuration was assigned to correspond with that of the known chiral centres in a precursor molecule, which remained unchanged during the synthesis of the title compound.

### Crystal data

**Table:**

C_24_H_30_N_2_O_4_	*F*(000) = 880
*M**_r_* = 410.50	*D*_x_ = 1.207 Mg m^−^^3^
Monoclinic, *C*2	Mo *K*α radiation, λ = 0.71073 Å
Hall symbol: C 2y	Cell parameters from 2344 reflections
*a* = 27.248 (4) Å	θ = 5.7–47.9°
*b* = 5.8241 (8) Å	µ = 0.08 mm^−^^1^
*c* = 14.275 (2) Å	*T* = 293 K
β = 94.192 (3)°	Prismatic, white
*V* = 2259.3 (5) Å^3^	0.37 × 0.24 × 0.16 mm
*Z* = 4	

### Data collection

**Table:**

Bruker SMART APEX CCD diffractometer	1934 reflections with *I* > 2σ(*I*)
Radiation source: fine-focus sealed tube	*R*_int_ = 0.110
graphite	θ_max_ = 25.5°, θ_min_ = 2.0°
φ and ω scans	*h* = −32→27
5956 measured reflections	*k* = −6→7
2310 independent reflections	*l* = −17→16

### Refinement

**Table:**

Refinement on *F*^2^	Primary atom site location: structure-invariant direct methods
Least-squares matrix: full	Secondary atom site location: difference Fourier map
*R*[*F*^2^ > 2σ(*F*^2^)] = 0.048	Hydrogen site location: inferred from neighbouring sites
*wR*(*F*^2^) = 0.109	H atoms treated by a mixture of independent and constrained refinement
*S* = 0.96	*w* = 1/[σ^2^(*F*_o_^2^) + (0.0542*P*)^2^] where *P* = (*F*_o_^2^ + 2*F*_c_^2^)/3
2310 reflections	(Δ/σ)_max_ = 0.018
279 parameters	Δρ_max_ = 0.15 e Å^−^^3^
1 restraint	Δρ_min_ = −0.23 e Å^−^^3^

### Special details

**Table:**

Geometry. All e.s.d.'s (except the e.s.d. in the dihedral angle between two l.s. planes) are estimated using the full covariance matrix. The cell e.s.d.'s are taken into account individually in the estimation of e.s.d.'s in distances, angles and torsion angles; correlations between e.s.d.'s in cell parameters are only used when they are defined by crystal symmetry. An approximate (isotropic) treatment of cell e.s.d.'s is used for estimating e.s.d.'s involving l.s. planes.
Refinement. Refinement of *F*^2^ against ALL reflections. The weighted *R*-factor *wR* and goodness of fit *S* are based on *F*^2^, conventional *R*-factors *R* are based on *F*, with *F* set to zero for negative *F*^2^. The threshold expression of *F*^2^ > σ(*F*^2^) is used only for calculating *R*-factors(gt) *etc*. and is not relevant to the choice of reflections for refinement. *R*-factors based on *F*^2^ are statistically about twice as large as those based on *F*, and *R*- factors based on ALL data will be even larger.

### Fractional atomic coordinates and isotropic or equivalent isotropic displacement parameters (Å^2^)

**Table:**

	*x*	*y*	*z*	*U*_iso_*/*U*_eq_	
N1	0.38365 (9)	0.0550 (5)	−0.09403 (17)	0.0726 (8)	
N2	0.32924 (10)	0.1668 (4)	0.19729 (14)	0.0582 (7)	
O1	0.36972 (10)	0.4751 (5)	0.15139 (14)	0.0892 (8)	
O2	0.30774 (6)	0.0299 (3)	0.02574 (11)	0.0516 (5)	
O3	0.39908 (8)	0.0609 (4)	−0.24661 (13)	0.0740 (6)	
O4	0.43729 (7)	0.3050 (4)	−0.14171 (13)	0.0649 (6)	
C1	0.34746 (10)	0.2991 (5)	0.13400 (17)	0.0504 (6)	
C2	0.34143 (9)	0.2148 (5)	0.03370 (16)	0.0484 (6)	
H2	0.3284	0.3407	−0.0063	0.058*	
C3	0.39051 (10)	0.1440 (7)	0.00143 (19)	0.0702 (9)	
H3A	0.4125	0.2750	0.0031	0.084*	
H3B	0.4051	0.0268	0.0429	0.084*	
C4	0.34907 (11)	−0.1318 (6)	−0.1033 (2)	0.0677 (8)	
H4A	0.3621	−0.2628	−0.0677	0.081*	
H4B	0.3443	−0.1769	−0.1687	0.081*	
C5	0.30039 (10)	−0.0621 (5)	−0.06807 (17)	0.0529 (7)	
H5	0.2804	−0.2014	−0.0645	0.064*	
C6	0.27212 (12)	0.1025 (7)	−0.13283 (19)	0.0747 (9)	
H6A	0.2429	0.1509	−0.1047	0.112*	
H6B	0.2632	0.0278	−0.1916	0.112*	
H6C	0.2922	0.2339	−0.1435	0.112*	
C7	0.33164 (10)	0.2209 (5)	0.29757 (15)	0.0528 (7)	
H7	0.3432	0.3797	0.3048	0.063*	
C8	0.28101 (10)	0.2103 (5)	0.33392 (16)	0.0512 (6)	
C9	0.25146 (10)	0.0219 (6)	0.31751 (19)	0.0610 (7)	
H9	0.2632	−0.1026	0.2850	0.073*	
C10	0.20460 (11)	0.0133 (7)	0.3483 (2)	0.0739 (9)	
H10	0.1847	−0.1143	0.3353	0.089*	
C11	0.18741 (13)	0.1950 (8)	0.3985 (2)	0.0795 (10)	
H11	0.1558	0.1915	0.4192	0.095*	
C12	0.21685 (14)	0.3780 (8)	0.4174 (2)	0.0825 (11)	
H12	0.2056	0.4985	0.4529	0.099*	
C13	0.26342 (13)	0.3900 (6)	0.38514 (18)	0.0684 (8)	
H13	0.2829	0.5189	0.3979	0.082*	
C14	0.36812 (9)	0.0700 (5)	0.35412 (16)	0.0486 (6)	
C15	0.38313 (10)	0.1338 (6)	0.44504 (18)	0.0625 (8)	
H15	0.3716	0.2702	0.4692	0.075*	
C16	0.41486 (12)	−0.0017 (8)	0.5002 (2)	0.0775 (10)	
H16	0.4245	0.0440	0.5612	0.093*	
C17	0.43244 (11)	−0.2042 (8)	0.4660 (3)	0.0787 (10)	
H17	0.4539	−0.2958	0.5033	0.094*	
C18	0.41775 (11)	−0.2685 (7)	0.3760 (2)	0.0744 (9)	
H18	0.4293	−0.4051	0.3521	0.089*	
C19	0.38604 (11)	−0.1329 (6)	0.32084 (19)	0.0606 (7)	
H19	0.3765	−0.1792	0.2599	0.073*	
C20	0.40627 (10)	0.1365 (5)	−0.16836 (19)	0.0562 (7)	
C21	0.47203 (10)	0.3996 (5)	−0.20592 (19)	0.0570 (7)	
C22	0.49962 (12)	0.5747 (6)	−0.1450 (2)	0.0765 (9)	
H22A	0.4769	0.6863	−0.1239	0.115*	
H22B	0.5235	0.6500	−0.1805	0.115*	
H22C	0.5160	0.4996	−0.0916	0.115*	
C23	0.50568 (12)	0.2101 (6)	−0.2339 (3)	0.0787 (10)	
H23A	0.5194	0.1329	−0.1786	0.118*	
H23B	0.5317	0.2743	−0.2674	0.118*	
H23C	0.4873	0.1025	−0.2735	0.118*	
C24	0.44503 (13)	0.5127 (7)	−0.2886 (2)	0.0795 (9)	
H24A	0.4274	0.3987	−0.3262	0.119*	
H24B	0.4681	0.5892	−0.3256	0.119*	
H24C	0.4222	0.6228	−0.2670	0.119*	
H2A	0.3113 (11)	0.060 (6)	0.181 (2)	0.058 (9)*	

### Atomic displacement parameters (Å^2^)

**Table:**

	*U*^11^	*U*^22^	*U*^33^	*U*^12^	*U*^13^	*U*^23^
N1	0.0724 (14)	0.096 (2)	0.0528 (13)	−0.0328 (16)	0.0287 (11)	−0.0251 (14)
N2	0.0865 (17)	0.0596 (16)	0.0294 (10)	−0.0232 (15)	0.0104 (10)	−0.0025 (11)
O1	0.1344 (19)	0.0868 (17)	0.0501 (11)	−0.0546 (17)	0.0324 (12)	−0.0172 (11)
O2	0.0594 (10)	0.0633 (12)	0.0333 (8)	−0.0150 (10)	0.0120 (7)	0.0012 (8)
O3	0.0932 (14)	0.0820 (16)	0.0498 (11)	−0.0177 (13)	0.0264 (10)	−0.0213 (11)
O4	0.0692 (11)	0.0794 (14)	0.0489 (10)	−0.0184 (12)	0.0226 (9)	−0.0078 (10)
C1	0.0606 (15)	0.0540 (16)	0.0374 (13)	−0.0084 (14)	0.0102 (11)	0.0000 (12)
C2	0.0617 (14)	0.0529 (15)	0.0318 (12)	−0.0124 (13)	0.0101 (10)	0.0041 (12)
C3	0.0651 (16)	0.104 (3)	0.0432 (14)	−0.0235 (18)	0.0182 (12)	−0.0143 (16)
C4	0.0791 (19)	0.066 (2)	0.0606 (17)	−0.0091 (17)	0.0238 (14)	−0.0143 (16)
C5	0.0658 (15)	0.0556 (17)	0.0388 (13)	−0.0199 (14)	0.0129 (11)	−0.0045 (12)
C6	0.086 (2)	0.091 (2)	0.0453 (15)	−0.010 (2)	−0.0075 (14)	−0.0075 (17)
C7	0.0807 (17)	0.0498 (15)	0.0284 (11)	−0.0124 (14)	0.0080 (11)	−0.0035 (12)
C8	0.0711 (16)	0.0536 (16)	0.0291 (11)	0.0047 (14)	0.0055 (11)	0.0035 (12)
C9	0.0695 (17)	0.0628 (19)	0.0515 (15)	0.0025 (17)	0.0093 (13)	−0.0056 (14)
C10	0.0677 (18)	0.091 (2)	0.0636 (18)	0.000 (2)	0.0090 (15)	0.0064 (19)
C11	0.073 (2)	0.104 (3)	0.0630 (19)	0.024 (2)	0.0158 (16)	0.008 (2)
C12	0.104 (3)	0.088 (3)	0.0577 (18)	0.035 (2)	0.0246 (17)	−0.002 (2)
C13	0.102 (2)	0.0628 (19)	0.0417 (14)	0.0082 (19)	0.0094 (15)	−0.0034 (15)
C14	0.0553 (13)	0.0569 (17)	0.0355 (12)	−0.0131 (13)	0.0173 (10)	−0.0044 (12)
C15	0.0768 (18)	0.072 (2)	0.0391 (13)	−0.0027 (17)	0.0048 (13)	−0.0101 (14)
C16	0.079 (2)	0.101 (3)	0.0518 (17)	−0.007 (2)	−0.0050 (15)	−0.0046 (19)
C17	0.0581 (17)	0.105 (3)	0.074 (2)	0.007 (2)	0.0088 (15)	0.018 (2)
C18	0.0694 (18)	0.078 (2)	0.079 (2)	0.0090 (18)	0.0257 (16)	0.0008 (19)
C19	0.0711 (17)	0.0677 (19)	0.0446 (14)	−0.0044 (16)	0.0152 (12)	−0.0072 (15)
C20	0.0553 (14)	0.0636 (17)	0.0517 (16)	−0.0059 (14)	0.0183 (12)	−0.0122 (14)
C21	0.0616 (16)	0.0558 (16)	0.0564 (15)	0.0001 (14)	0.0221 (12)	0.0089 (14)
C22	0.0780 (19)	0.074 (2)	0.079 (2)	−0.0112 (19)	0.0170 (16)	0.0076 (19)
C23	0.0762 (19)	0.067 (2)	0.097 (3)	0.0062 (19)	0.0353 (17)	0.007 (2)
C24	0.102 (2)	0.071 (2)	0.0663 (19)	0.007 (2)	0.0086 (17)	0.0084 (17)

### Geometric parameters (Å, °)

**Table:**

N1—C20	1.352 (4)	C9—H9	0.9300
N1—C4	1.439 (4)	C10—C11	1.379 (5)
N1—C3	1.457 (3)	C10—H10	0.9300
N2—C1	1.312 (3)	C11—C12	1.349 (6)
N2—C7	1.463 (3)	C11—H11	0.9300
N2—H2A	0.81 (3)	C12—C13	1.383 (5)
O1—C1	1.207 (4)	C12—H12	0.9300
O2—C2	1.414 (3)	C13—H13	0.9300
O2—C5	1.443 (3)	C14—C19	1.376 (4)
O3—C20	1.203 (3)	C14—C15	1.383 (3)
O4—C20	1.332 (3)	C15—C16	1.375 (5)
O4—C21	1.472 (3)	C15—H15	0.9300
C1—C2	1.511 (3)	C16—C17	1.376 (6)
C2—C3	1.503 (4)	C16—H16	0.9300
C2—H2	0.9800	C17—C18	1.369 (5)
C3—H3A	0.9700	C17—H17	0.9300
C3—H3B	0.9700	C18—C19	1.376 (4)
C4—C5	1.508 (4)	C18—H18	0.9300
C4—H4A	0.9700	C19—H19	0.9300
C4—H4B	0.9700	C21—C24	1.497 (4)
C5—C6	1.504 (4)	C21—C22	1.505 (5)
C5—H5	0.9800	C21—C23	1.507 (4)
C6—H6A	0.9600	C22—H22A	0.9600
C6—H6B	0.9600	C22—H22B	0.9600
C6—H6C	0.9600	C22—H22C	0.9600
C7—C8	1.510 (4)	C23—H23A	0.9600
C7—C14	1.515 (4)	C23—H23B	0.9600
C7—H7	0.9800	C23—H23C	0.9600
C8—C9	1.371 (4)	C24—H24A	0.9600
C8—C13	1.382 (4)	C24—H24B	0.9600
C9—C10	1.382 (4)	C24—H24C	0.9600
			
C20—N1—C4	121.8 (2)	C9—C10—H10	120.2
C20—N1—C3	125.1 (3)	C12—C11—C10	119.4 (3)
C4—N1—C3	113.2 (2)	C12—C11—H11	120.3
C1—N2—C7	123.7 (2)	C10—C11—H11	120.3
C1—N2—H2A	120 (2)	C11—C12—C13	121.3 (3)
C7—N2—H2A	115 (2)	C11—C12—H12	119.4
C2—O2—C5	113.74 (17)	C13—C12—H12	119.4
C20—O4—C21	121.4 (2)	C8—C13—C12	120.0 (4)
O1—C1—N2	124.5 (2)	C8—C13—H13	120.0
O1—C1—C2	119.3 (2)	C12—C13—H13	120.0
N2—C1—C2	116.1 (2)	C19—C14—C15	117.8 (3)
O2—C2—C3	110.7 (2)	C19—C14—C7	123.3 (2)
O2—C2—C1	110.40 (19)	C15—C14—C7	118.8 (2)
C3—C2—C1	110.0 (2)	C16—C15—C14	120.9 (3)
O2—C2—H2	108.6	C16—C15—H15	119.6
C3—C2—H2	108.6	C14—C15—H15	119.6
C1—C2—H2	108.6	C15—C16—C17	120.6 (3)
N1—C3—C2	109.1 (2)	C15—C16—H16	119.7
N1—C3—H3A	109.9	C17—C16—H16	119.7
C2—C3—H3A	109.9	C18—C17—C16	118.8 (3)
N1—C3—H3B	109.9	C18—C17—H17	120.6
C2—C3—H3B	109.9	C16—C17—H17	120.6
H3A—C3—H3B	108.3	C17—C18—C19	120.6 (3)
N1—C4—C5	110.6 (3)	C17—C18—H18	119.7
N1—C4—H4A	109.5	C19—C18—H18	119.7
C5—C4—H4A	109.5	C18—C19—C14	121.3 (3)
N1—C4—H4B	109.5	C18—C19—H19	119.4
C5—C4—H4B	109.5	C14—C19—H19	119.4
H4A—C4—H4B	108.1	O3—C20—O4	126.3 (3)
O2—C5—C6	111.3 (2)	O3—C20—N1	123.2 (3)
O2—C5—C4	110.1 (2)	O4—C20—N1	110.5 (2)
C6—C5—C4	112.9 (2)	O4—C21—C24	110.7 (2)
O2—C5—H5	107.4	O4—C21—C22	102.1 (2)
C6—C5—H5	107.4	C24—C21—C22	110.7 (3)
C4—C5—H5	107.4	O4—C21—C23	108.8 (2)
C5—C6—H6A	109.5	C24—C21—C23	112.8 (3)
C5—C6—H6B	109.5	C22—C21—C23	111.2 (3)
H6A—C6—H6B	109.5	C21—C22—H22A	109.5
C5—C6—H6C	109.5	C21—C22—H22B	109.5
H6A—C6—H6C	109.5	H22A—C22—H22B	109.5
H6B—C6—H6C	109.5	C21—C22—H22C	109.5
N2—C7—C8	110.5 (2)	H22A—C22—H22C	109.5
N2—C7—C14	112.2 (2)	H22B—C22—H22C	109.5
C8—C7—C14	111.9 (2)	C21—C23—H23A	109.5
N2—C7—H7	107.3	C21—C23—H23B	109.5
C8—C7—H7	107.3	H23A—C23—H23B	109.5
C14—C7—H7	107.3	C21—C23—H23C	109.5
C9—C8—C13	118.3 (3)	H23A—C23—H23C	109.5
C9—C8—C7	120.9 (2)	H23B—C23—H23C	109.5
C13—C8—C7	120.8 (3)	C21—C24—H24A	109.5
C8—C9—C10	121.3 (3)	C21—C24—H24B	109.5
C8—C9—H9	119.4	H24A—C24—H24B	109.5
C10—C9—H9	119.4	C21—C24—H24C	109.5
C11—C10—C9	119.6 (4)	H24A—C24—H24C	109.5
C11—C10—H10	120.2	H24B—C24—H24C	109.5
			
C7—N2—C1—O1	2.9 (5)	C9—C10—C11—C12	−0.5 (5)
C7—N2—C1—C2	−180.0 (2)	C10—C11—C12—C13	1.9 (5)
C5—O2—C2—C3	−57.3 (3)	C9—C8—C13—C12	−0.8 (4)
C5—O2—C2—C1	−179.4 (2)	C7—C8—C13—C12	179.6 (2)
O1—C1—C2—O2	−170.2 (3)	C11—C12—C13—C8	−1.3 (5)
N2—C1—C2—O2	12.4 (3)	N2—C7—C14—C19	−17.3 (3)
O1—C1—C2—C3	67.3 (4)	C8—C7—C14—C19	107.6 (3)
N2—C1—C2—C3	−110.0 (3)	N2—C7—C14—C15	165.0 (2)
C20—N1—C3—C2	123.2 (3)	C8—C7—C14—C15	−70.1 (3)
C4—N1—C3—C2	−56.1 (4)	C19—C14—C15—C16	−0.1 (4)
O2—C2—C3—N1	55.5 (3)	C7—C14—C15—C16	177.8 (3)
C1—C2—C3—N1	177.8 (3)	C14—C15—C16—C17	0.0 (5)
C20—N1—C4—C5	−124.0 (3)	C15—C16—C17—C18	0.0 (5)
C3—N1—C4—C5	55.3 (4)	C16—C17—C18—C19	0.1 (5)
C2—O2—C5—C6	−70.7 (3)	C17—C18—C19—C14	−0.2 (4)
C2—O2—C5—C4	55.4 (3)	C15—C14—C19—C18	0.1 (4)
N1—C4—C5—O2	−52.7 (3)	C7—C14—C19—C18	−177.6 (3)
N1—C4—C5—C6	72.4 (3)	C21—O4—C20—O3	−8.3 (4)
C1—N2—C7—C8	126.8 (3)	C21—O4—C20—N1	170.6 (3)
C1—N2—C7—C14	−107.5 (3)	C4—N1—C20—O3	0.4 (5)
N2—C7—C8—C9	51.2 (3)	C3—N1—C20—O3	−178.8 (3)
C14—C7—C8—C9	−74.6 (3)	C4—N1—C20—O4	−178.5 (3)
N2—C7—C8—C13	−129.2 (3)	C3—N1—C20—O4	2.3 (4)
C14—C7—C8—C13	105.0 (3)	C20—O4—C21—C24	63.9 (4)
C13—C8—C9—C10	2.1 (4)	C20—O4—C21—C22	−178.2 (2)
C7—C8—C9—C10	−178.2 (2)	C20—O4—C21—C23	−60.5 (3)
C8—C9—C10—C11	−1.5 (4)		

### Hydrogen-bond geometry (Å, °)

**Table:**

*D*—H···*A*	*D*—H	H···*A*	*D*···*A*	*D*—H···*A*
C19—H19···O1^i^	0.93	2.54	3.332 (4)	144

Symmetry codes: (i) *x*, *y*−1, *z*.
